# Many-body effects in semiconducting single-wall silicon nanotubes

**DOI:** 10.3762/bjnano.5.2

**Published:** 2014-01-06

**Authors:** Wei Wei, Timo Jacob

**Affiliations:** 1 Institute of Electrochemistry, Ulm University, Albert-Einstein-Allee 47, D-89081 Ulm, Germany

**Keywords:** Bethe–Salpeter equation, excitons, *GW* approximation, many body effects, silicon

## Abstract

The electronic and optical properties of semiconducting silicon nanotubes (SiNTs) are studied by means of the many-body Green’s function method, i.e., *GW* approximation and Bethe–Salpeter equation. In these studied structures, i.e., (4,4), (6,6) and (10,0) SiNTs, self-energy effects are enhanced giving rise to large quasi-particle (QP) band gaps due to the confinement effect. The strong electron−electron (*e*−*e*) correlations broaden the band gaps of the studied SiNTs from 0.65, 0.28 and 0.05 eV at DFT level to 1.9, 1.22 and 0.79 eV at *GW* level. The Coulomb electron−hole (*e*−*h*) interactions significantly modify optical absorption properties obtained at noninteracting-particle level with the formation of bound excitons with considerable binding energies (of the order of 1 eV) assigned: the binding energies of the armchair (4,4), (6,6) and zigzag (10,0) SiNTs are 0.92, 1.1 and 0.6 eV, respectively. Results in this work are useful for understanding the physics and applications in silicon-based nanoscale device components.

## Introduction

Silicon nanotubes [1–5] (SiNTs) have been demonstrated to be emerging materials with exclusive applications in micro- and nanoelectronics [6–12]. An extra advantage of SiNTs lies in the natural compatibility with current silicon-based technology. Their noncytotoxic nature, further, makes them promising candidates for a large variety of biotechnological applications as well. In addition, due to the quantum confinement effect, SiNTs have great potential for photoemission applications directly on silicon substrates that in turn could lead to the integration of photonics and microelectronic devices on a single chip [13]. One-dimensional SiNTs have been viewed as potential basic building blocks for future applications. SiNTs exhibit, for instance, excellent electrochemical performances when being used as anodes for lithium rechargeable batteries [10–12]. Although the ground-state geometric and electronic properties of SiNTs have been studied [14–17], properties of excited states, for example optical absorption of SiNTs, are still in need. It is of high importance to correctly understand the optical properties of SiNTs due to fundamental applications in electro-optical fields.

In structures with reduced dimensionality, higher quasi-particle (QP) excitation energies can be reached by confinement effects, which enhance electron−electron (*e*−*e*) self-energy effects. In addition, reduced electronic screening leads to the formation of excitonic resonances or strongly bound excitons with considerable binding energies. Therefore, many-body effects [18–31] are required to understand this kind of systems sufficiently, especially their single-particle excitation and optical absorption properties.

It is well-known that density functional theory (DFT) often fails in describing the properties of light absorption. This process requires a description of two-particle properties, which certainly goes beyond single-electron excitations that can be described at the purely electronic level. Fortunately it is possible to make quantitative predictions of the absorption spectra and the band structures of a wide class of systems by combining the *GW* approximation and the Bethe−Salpeter equation (BSE) [32–37], i.e., many-body Green’s function perturbation theory. The *GW*+BSE scheme properly includes the *e−e* correlations and *e−h* interactions and its results frequently match experimental results in an excellent manner [38–41].

**Figure 1:**
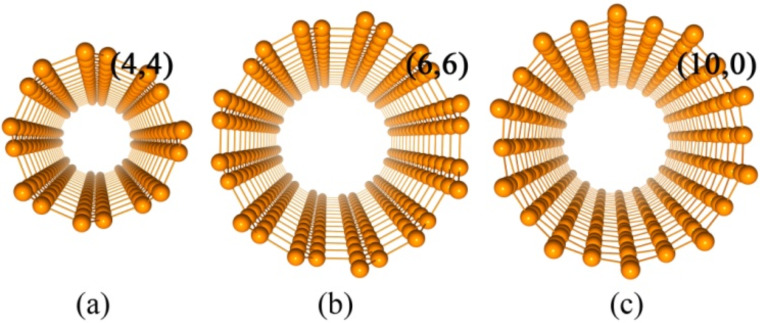
Perspective view of silicon nanotubes (4,4) (a), (6,6) (b) and (10,0) (c).

In the present work, many-body effects in semiconducting single-wall (4,4), (6,6) and (10,0) SiNTs, as shown in Figure 1, are studied. It has been identified that the self-energy effects are evident in the studied SiNTs, giving rise to large QP band gaps, and the excitonic effects distinctly modify optical absorption properties, resulting in the formation of bound excitons with considerable binding energies. The results shed some light on understanding the physical properties of SiNTs and potential applications in silicon-based nanoscale device components. It should be pointed out that many-body effects have also been highlighted in silicon nanowires [24,42] and carbon nanotubes due to the reduced dimensionality [43–45].

## Computational details

On the basis of DFT-calculated ground-state wave functions, obtained with the plane wave packge QUANTUM ESPRESSO [46], QP calculations have been performed. For these calculations we used a plane-wave basis set with a cutoff energy of 50 Ry and the local density approximation (LDA) for the exchange−correlation energy in conjunction with the norm-conserving pseudopotentials for treating the core-electrons. A Monkhorst−Pack ***k***-mesh of 20 integration points is used along the SiNTs tubes. A sufficiently large vacuum spacing of 13 Å surrounding the tube is imposed to separate periodic images and to avoid spurious interactions. Geometry optimization has been done with an energy convergence criterion of 5.0 × 10^−6^ eV and a force convergence criterion of 0.01 eV/Å.

As already summarized in [47], starting from the LDA wave functions and Coulomb screening, QP energies (within the *GW* approximation for the electron self-energy operator Σ) are obtained by solving the Dyson equation [48]:


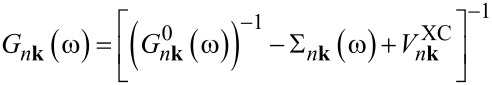


with the non-interacting Green’s function


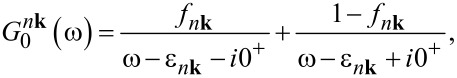


and *f*_n_**_k_** being the occupation factor and ε_n_**_k_** the Kohn−Sham energies. The Dyson equation is solved non-self-consistently, i.e., within the *G*_0_*W*_0_ approximation, leading to Σ = i*G*_0_*W*_0_. Though a fully self-consistent *GW* calculation often improves the accuracy, in some cases it can even overestimate the band gap. As the *G*_0_*W*_0_ approximation gives a band gap for bulk Si in excellent agreement with experiment, no self-consistent *GW*-treatment was necessary in the present work. Afterwards the random-phase approximation (RPA) was employed to obtain the reducible response function, while the generalized plasmon−pole model served as basis for treating dynamical screening effects in the self-energy.

The optical absorption spectrum, which is directly associated with the imaginary part of the macroscopic dielectric function, is defined in terms of the microscopic inverse dielectric function as


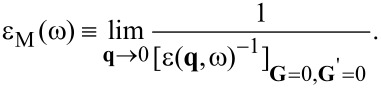


The coupled excitonic effects and absorption spectra are calculated by solving the BSE in terms of two-particle Green’s function of quasi-electron and quasi-hole states, which is obtained by performing a second iteration of Hedin’s equation:


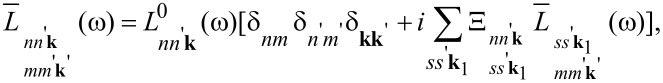


where 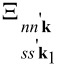 is the kernel describing tow-particle screened interactions. 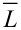 satisfies the following relation:


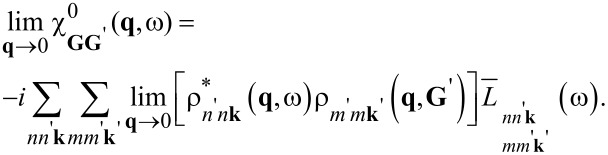


Finally, the macroscopic dielectric function can be expressed as


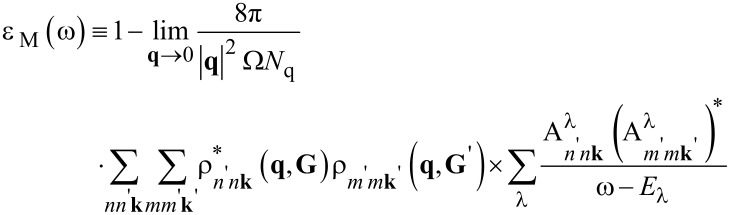


The standard Tamm−Dancoff approximation is adopted, in which only the positive *e*−*h* interactions are considered and, in addition, the non-Hermitian BSE reduces to a Hermitian one that can be solved with efficient and stable iterative methods [49]. For studying excitations in nanostructures [50] and in molecular systems [51] solving the BSE within the Tamm−Dancoff approximation is successively becoming a standard tool. For both the *GW* and BSE calculations, a box-shaped truncation of 13 Å is applied to screen the Coulomb interactions. Calculations including many-body effects are performed by using the YAMBO program suite [48].

## Results and Discussion

On the DFT-LDA level, the band gap of Si is calculated to be 0.61 eV, while at the *GW* level it turns out to be 1.17 eV, in good accordance to the band gap obtained from experiment [52]. As demonstrated in previous theoretical works [25,33], we reproduce the optical absorption spectrum of bulk Si with excitonic effects emphasized. The analogue of graphene but with Si instead of C is two-dimensional silicene, for which many efforts have been made to synthesize this material [53–54]. In silicene, massless Dirac fermions, as in graphene, have been demonstrated, and thus silicene holds a substantial promise for future applications in nanoelectronics. Because of the presence of two-dimensional silicene, the synthesis of one-dimensional SiNTs is waiting for its realization. It has been discussed that the extra cost to produce SiNTs from silicene is of the same order of the equivalent cost in carbon [1]. In silicene, the optical responses are characterized by resonant excitations [54].

**Figure 2:**
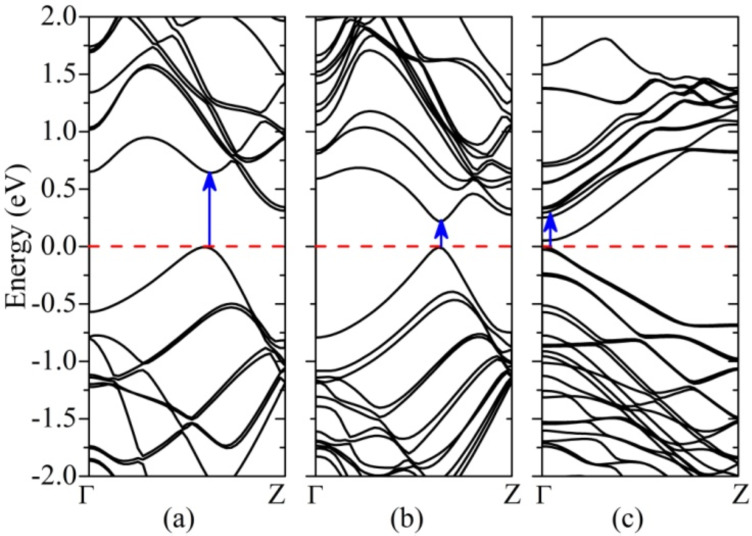
Band structures of silicon nanotubes (4,4) (a), (6,6) (b) and (10,0) (c). The Fermi level is set at the top of valence band, and the arrows indicate the *k* point where the inter band transitions occur.

Figure 2 shows the band structures of (4,4), (6,6) and (10,0) SiNTs, which indicate semiconducting character as band gaps appear. In case of armchair (4,4) SiNT, the band structure indicates an indirect band gap of 0.32 eV at Z point and a direct band gap of 0.65 eV at the *k* point on two-thirds way from Γ to Z in the one-dimensional Brillouin zone along the tube (2π/3a). However, it has been indicated that (*n*,*n*) armchair SiNTs are not stable for *n* < 6 [14]. In the case of a more stable armchair (6,6) and zigzag (10,0) SiNTs, as-calculated band structures show direct band gaps of 0.28 and 0.05 eV (50 meV), respectively. In (10,0) SiNT, the vanishingly narrow gap appears at the Γ point. However, there are still controversies with respect to the dependence of the conductance (semiconducting or metallic) on the chirality and/or diameter of SiNTs [1,14–15]. This discrepancy can be attributed, for instance, to the different symmetries of the initial structures from where SiNTs are constructed [15]. In the current work, after geometry relaxation, SiNTs prefer to be in a gear-like configuration.

However, the standard DFT-based method generally underestimates band gaps because the independent particle picture breaks down. When including the *e*−*e* self-energy effects within the *GW* approximation, band gaps of the selected SiNTs are significantly broadened to be 1.9 (1.43 for indirect gap), 1.22 and 0.79 eV for the different SiNTs, respectively. In Table 1, all the relevant values are summarized. In the framework of *GW* approximation, such enormous modifications on the Kohn−Sham LDA values originate from enhanced *e*−*e* correlations due to the confinement effect in SiNTs with reduced dimensionality. A simple “scissor rule” is inapplicable due to the fact that the screening behavior determines the QP corrections [55]. For example, the QP self-energy effects give electron and hole masses smaller than the values predicted by DFT [56]. The nonlocal character of the self-energy operator in the *GW* framework is responsible for such a behavior [57]. As illustrated later, the exciton binding energy compensates the discrepancy between the electronic band gap calculated by DFT and the optical gap observed experimentally.

**Table 1:** Band gap at DFT-LDA and *GW* levels (*E*_g-DFT_ and *E*_g-_*_GW_*), QP corrections to the DFT-LDA gaps (QPC), excitation energy (*E*^I^) and binding energy (*E*^b^) of the first bound exciton of silicon nanotubes. All values are in eV.

	*E*_g-DFT_	*E*_g-_*_GW_*	QPC	*E*^I^	*E*^b^

(4,4)	0.65	1.9	1.25	0.98	0.92
(6,6)	0.28	1.22	0.94	0.12	1.1
(10,0)	0.05	0.79	0.74	0.19	0.6

**Figure 3:**
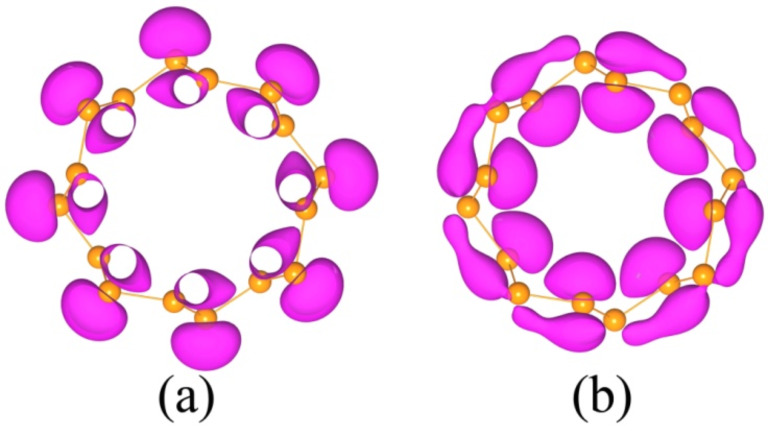
Charge density of the last valence band (a) and the first conduction band (b) of (4,4) SiNT at the *k* point at which point direct transitions occurrs (see arrow in Figure 2a).

As a representation, electronic wave functions of the last valence band and the first conduction band of (4,4) SiNT at the *k* point at which the direct transition occurrs are shown in Figure 3. As can be seen from the wave function of the last valence band, the weak π bonds are predominating with *p*_z_ states floating above Si atoms due to the fact that *sp^3^* hybridization in silicon is stable, which is in accordance with the mixed *sp^2^*–*sp^3^* hybridization in silicene. In Figure 3b, a strong mixture of the π* states and σ* states exists in the tubes forming a ring-like distribution due to the curvature effects. In addition, one can see where the electrons are excited (with holes left).

**Figure 4:**
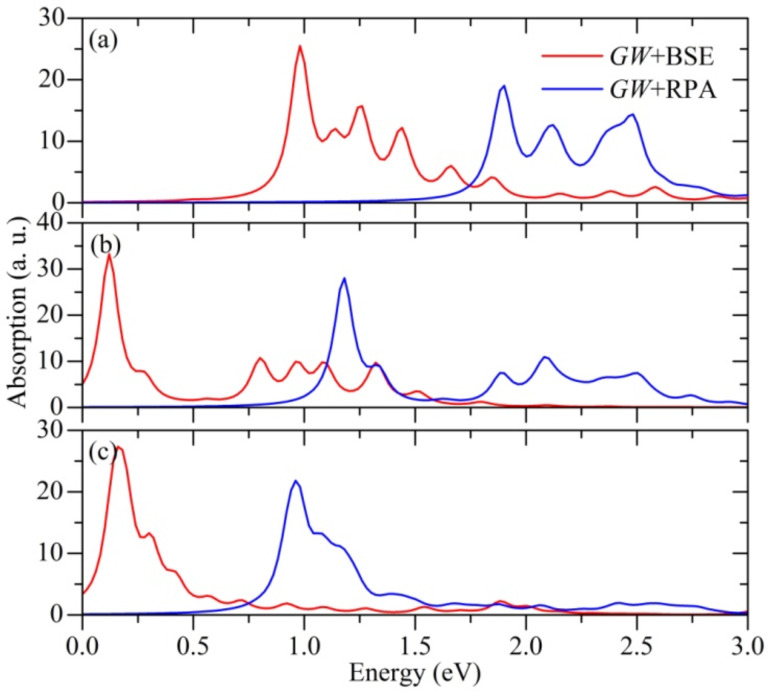
Absorption spectra of silicon nanotubes for light polarization along the tube calculated with and without the inclusion of *e*−*h* Coulomb interactions, i.e., *GW*+BSE and *GW*+RPA, respectively: (4,4) (a), (6,6) (b) and (10,0) (c). For the *GW*+BSE calculation, five occupied and five empty bands and a Lorentzian broadening of 0.05 eV are used.

In Figure 4, optical absorption spectra of studied SiNTs for light propagation along the tube are presented. In case of SiNTs, the quasi-one-dimensional nature causes optical transitions to obey well-defined selection rules across the entire bands. In comparison with the spectra at independent-particle level (LDA-RPA, not shown), self-energy effects generally blue-shift the oscillator strength (*GW*+RPA). When considering *e*−*h* interactions (*GW*+BSE), optical absorption properties of SiNTs are characterized by strong excitonic effects. In comparison with the single-particle spectrum, as can be seen from Figure 4, this prominent variation in weight redistribution of the oscillator strength reveals a global red-shift of the whole spectrum. In particular, optically active excitons (bound) below the onset of the single-particle transition continuum turns up with considerably large binding energies. The excitation energies *E*^I^ of the first bound excitons (the first sharp peaks in Figure 4) of (4,4), (6,6) and (10,0) SiNTs are 0.98, 0.12 and 0.19 eV, respectively. Binding energies, defined as the differences between the excitonic energy and the one-particle continuum onset and can be considered as a sign of *e*−*h* interactions, of these bound excitons are 0.92, 1.1 and 0.6 eV for (4,4), (6,6) and (10,0) SiNTs, respectively. The optically allowed transitions are mainly ascribed to the direct transitions between the last valence band and the first conduction band in (4,4) and (6,6) SiNTs, and between the last valence band and the third conduction band in the case of (10,0) SiNT. The smaller binding energy of the bright exciton in (10,0) SiNT is probably a consequence of the smaller quasi-electron effective mass. Since the interband transitions between the last valence band and the first conduction band are dipole-forbidden, there are several dark (optically inactive) excitons below the bound exciton in (10,0) SiNT. Compared with two-dimensional silicene, excitonic effects in SiNTs are stronger due to the lower dimensionality.

**Figure 5:**
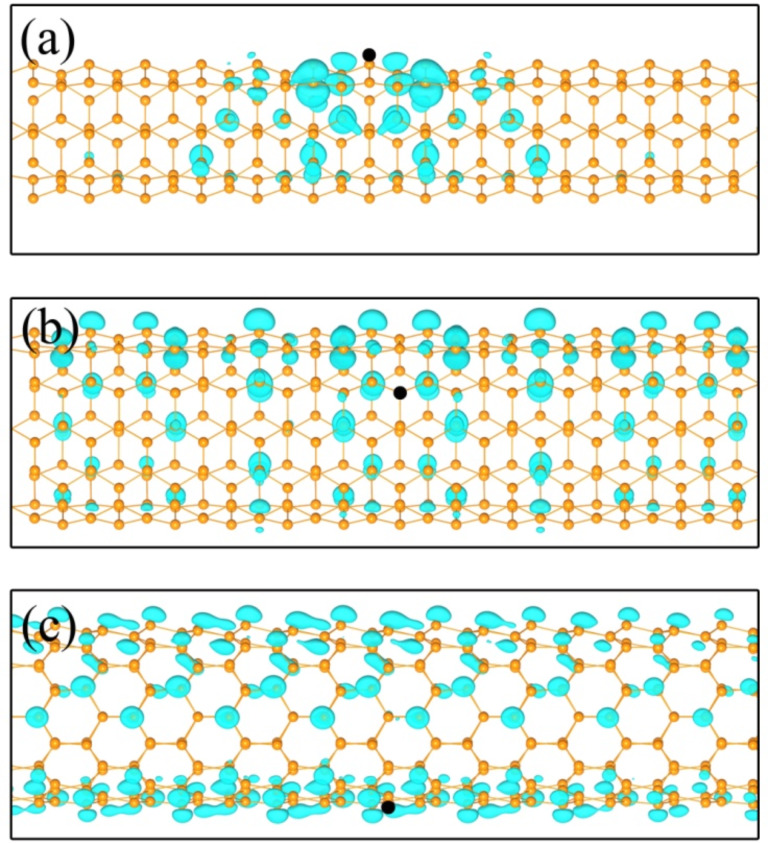
Electron probability distribution |*ψ*(***r****_e_*;***r****_h_*)|^2^ for finding the electrons ***r****_e_* with the hole position **r***_h_* (black dot) fixed slightly above a Si atom for the bound excitonic state of silicon nanotubes (4,4) (a); (6,6) (b) and (10,0) (c).

The BS two-particle’s Hamiltonian was diagonalized to obtain the *e*−*h* wave functions (quantum amplitudes) to show the correlation between excited electrons and holes in real space. Figure 5 shows the resulting three-dimensional electron probability distribution |ψ(***r****_e_*;***r****_h_*)|^2^ of the bound excitons of selected SiNTs. As shown in Figure 5a, the bound exciton of (4,4) SiNT has a relatively small distribution radius featured by a damping nature, which is an indication of a strong binding between excited electrons and holes with large binding energy. In the case of (6,6) and (10,0) SiNTs, the wave functions of the first bound excitons extend far away along the tubes, similar to a nature of resonant excitons. The reduced electronic screening governs the strong binding of excitons in SiNTs, which is revealed by the huge overlap of exciton wave functions. In all studied SiNTs structures, exciton wave functions are strongly cylindrically asymmetric.

**Figure 6:**
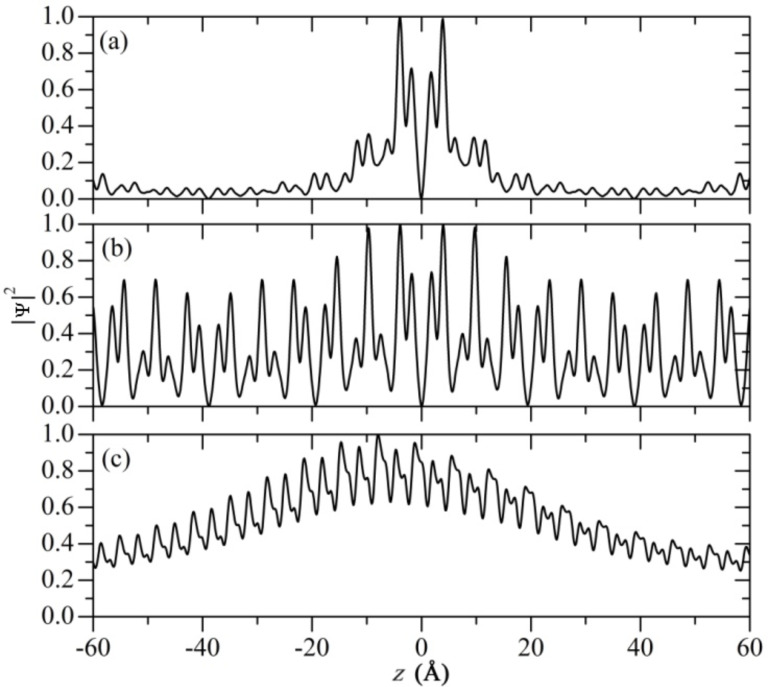
One-dimensional electron probability distribution |Ψ(***r****_e_*;***r****_h_*)|^2^ in real space of the first excitonic state (the first absorption peak) of silicon nanotubes (4,4) (a), (6,6) (b) and (10,0) (c). This one-dimensional distribution is plotted along the tube axis (*z*) with the hole position ***r****_h_* fixed at zero; the other coordinates are integrated out.

Quantitative representations of the electron distribution of the first excitons of studied SiNTs are demonstrated in Figure 6. In agreement with the exciton wave functions shown in Figure 5, the first bound exciton of (4,4) SiNTs is mainly localized within a radius of 20 Å. In the case of (6,6) and (10,0) SiNTs, the exciton radii extend over 60 Å. However, we also can see the damping nature, and the envelope function in the case of (10,0) SiNT.

## Conclusion

In summary, electronic and optical properties of single-wall semiconducting SiNTs have been studied by means of first-principles many-body perturbation theory. It has been elucidated that many-body effects strongly depend on the dimensionality of the system, and the quasi-one-dimensional features can be reflected in band structures and optical responses. One-dimensional structures, armchair (4,4) and (6,6), and zigzag (10,0) SiNTs, have been proven to be characterized by strong many-body effects due to the quantum confinement effect. The self-energy effects increase the single-particle excitation energy resulting in large QP band gaps in the studied SiNTs. The absorption properties are dominated by excitonic effects due to the reduced electronic screening giving rise to the formation of bound excitons with considerable binding energy. Since SiNTs are of great interest for basic scientific studies as well as potential applications, results in this work are of importance for a good understanding such systems.
